# Supplementary figures and images for: Differentiation Disorders of Chara vulgaris Spermatids following Treatment with Propyzamide
Source: Cells. 2023 Apr 27;12(9):1268. doi: 10.3390/cells12091268 (PMC10177507; doi:10.3390/cells12091268)

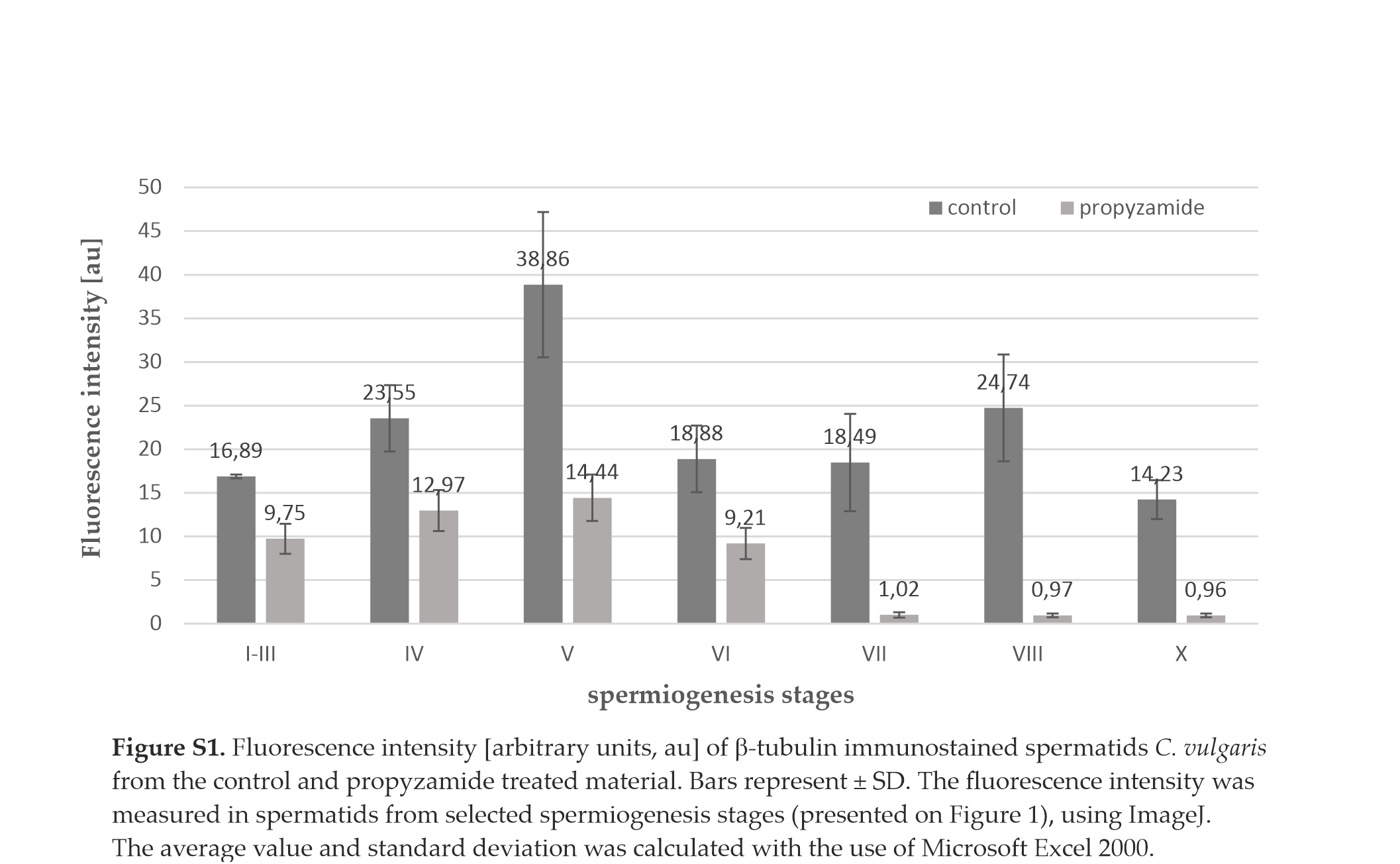

Supplement: Supplementary file 1 [file cells-12-01268-s001.zip › Fig. S1.tif]

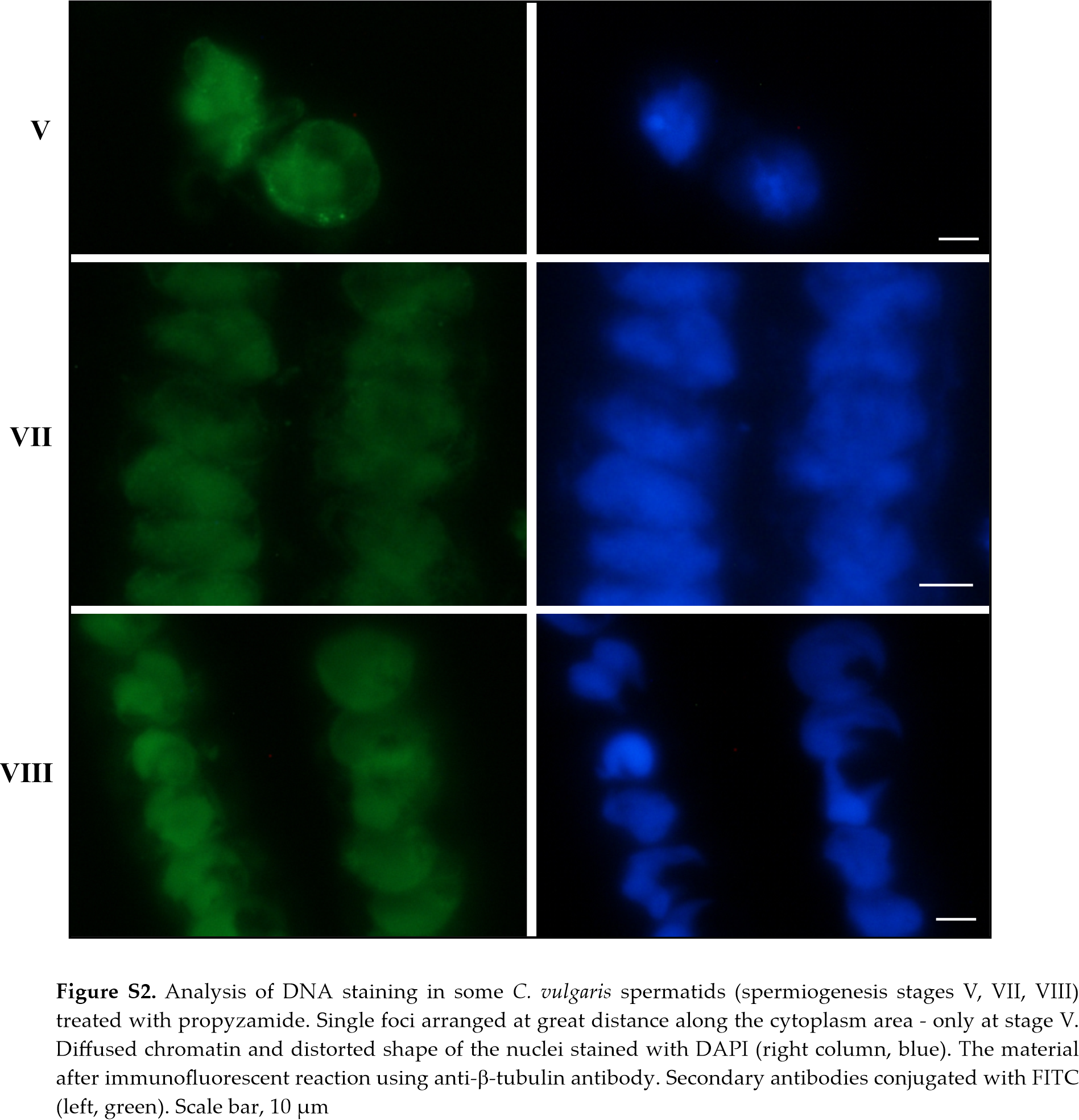

Supplement: Supplementary file 1 [file cells-12-01268-s001.zip › Fig. S2.tif]

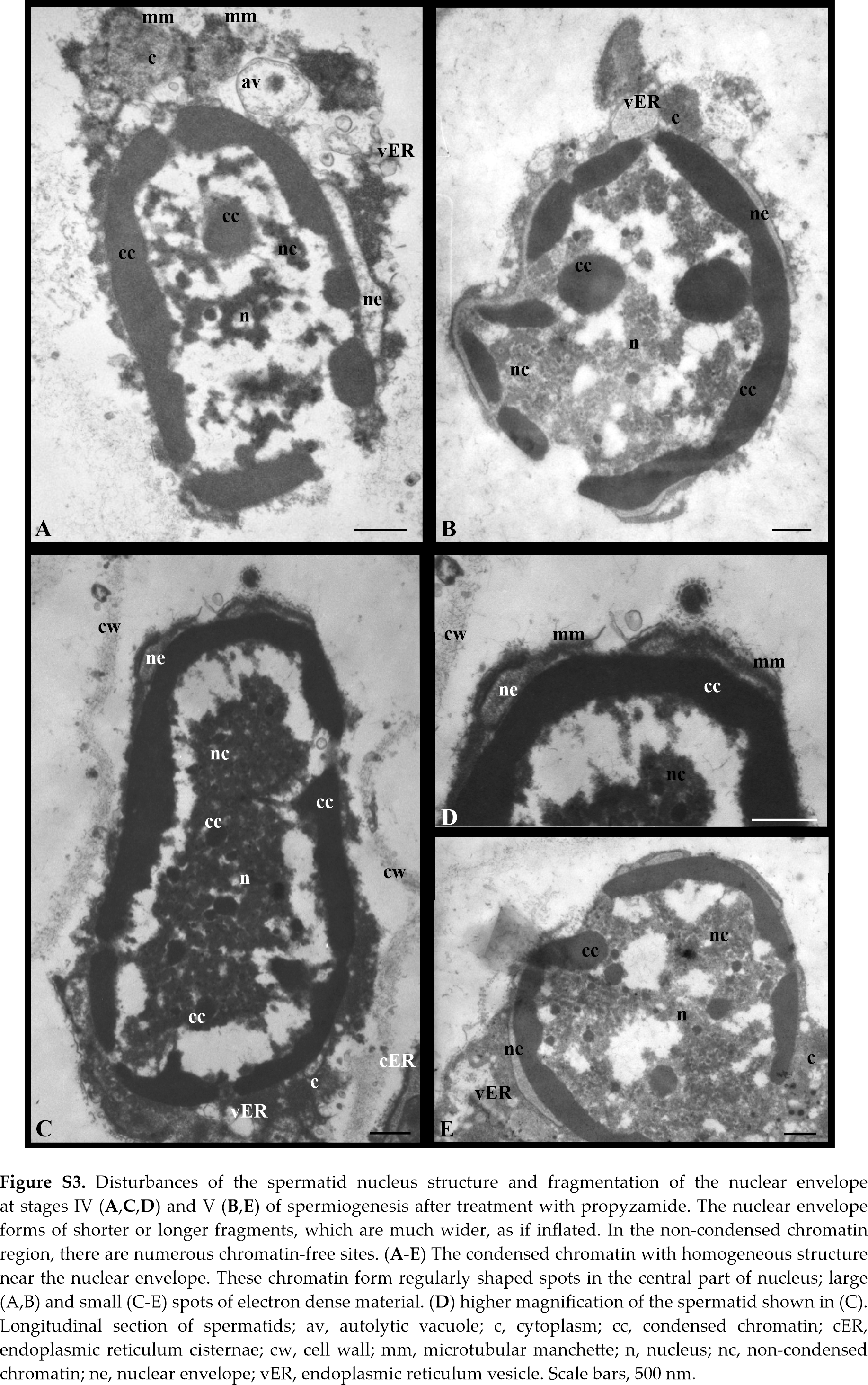

Supplement: Supplementary file 1 [file cells-12-01268-s001.zip › Fig. S3.tif]

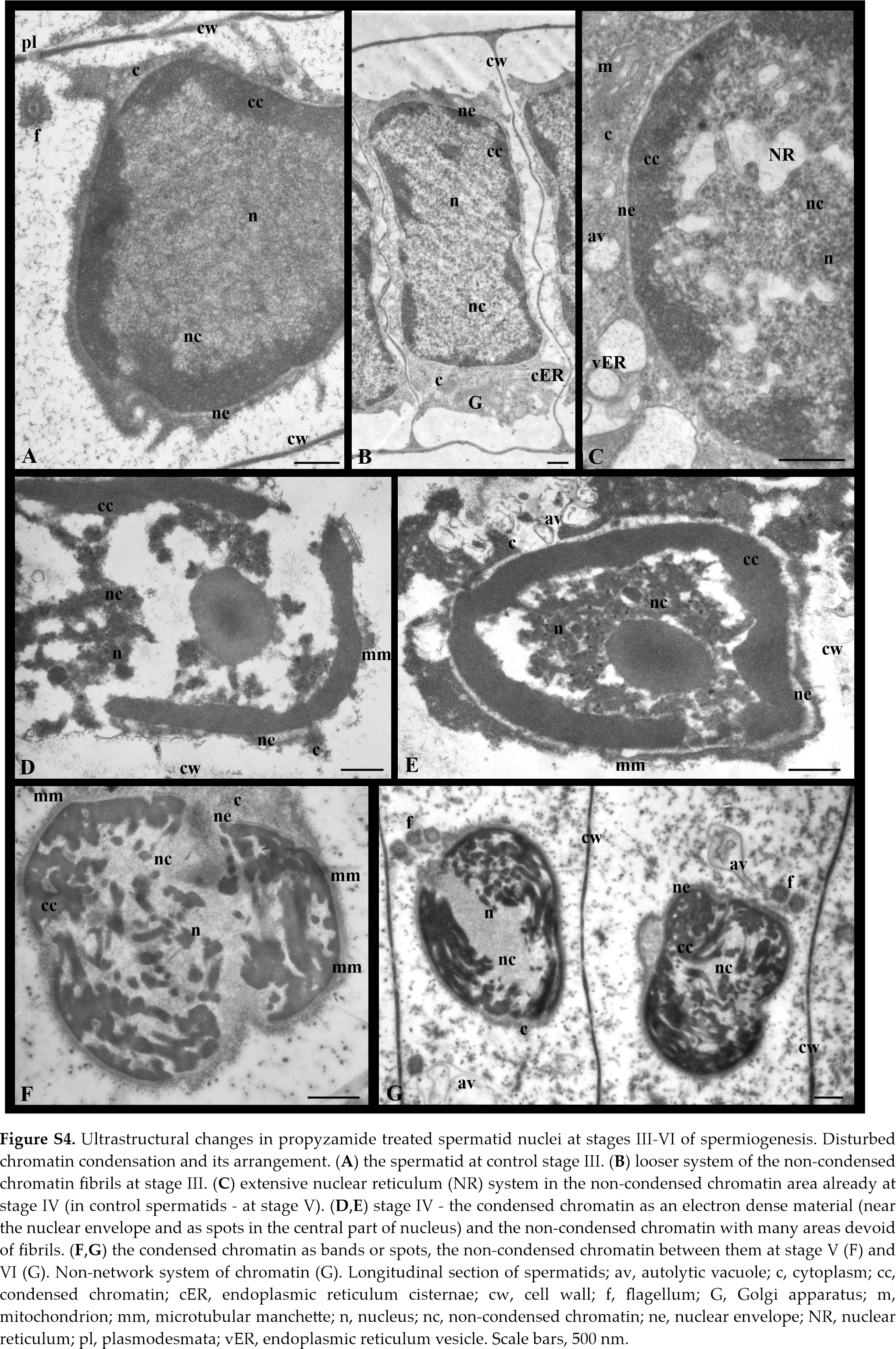

Supplement: Supplementary file 1 [file cells-12-01268-s001.zip › Fig. S4.tif]

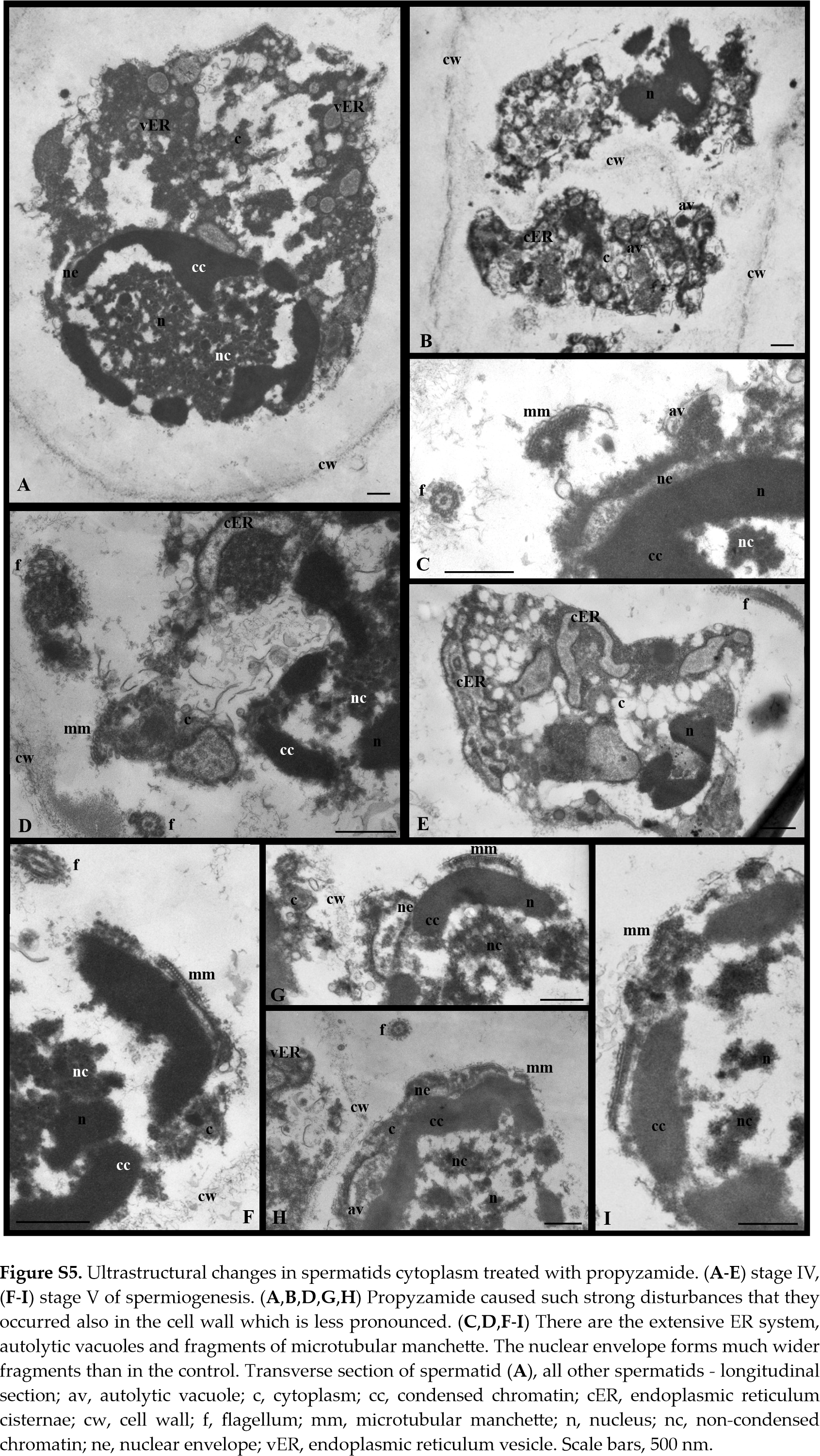

Supplement: Supplementary file 1 [file cells-12-01268-s001.zip › Fig. S5.tif]
